# Minimising feeding behaviour interference: A hay‐shaker device to assess dust exposure in horses

**DOI:** 10.1111/evj.14492

**Published:** 2025-03-03

**Authors:** Virginie Marie Angèle Bouverat, Jan Naef, Gaudenz Dolf, Inès Lamon, Sophie Elena Sage, Vinzenz Gerber

**Affiliations:** ^1^ Swiss Institute of Equine Medicine (ISME), Department of Clinical Veterinary Medicine, Vetsuisse‐Faculty University of Bern Bern Switzerland; ^2^ Agroscope, Swiss National Stud Farm Les Longs‐Prés Avenches Switzerland

**Keywords:** airborne irritants, environmental exposure, forage, horse, particulate matter

## Abstract

**Background:**

Organic dust from hay is a primary trigger of equine asthma.

**Objectives:**

(1) To introduce a novel hay‐shaker (HS) device for standardised hay dust generation, enabling simultaneous measurements of various particulate matter (PM) size fractions. (2) To compare these measurements with those in the horse's breathing zone (BZ) to assess the influence of environmental and individual factors.

**Study Design:**

Comparative experimental study.

**Methods:**

A HS generating dust was developed. Total dust (PMT) and size fractions (PM1, PM2.5, PM4 and PM10, representing particle size in μm) were measured from 50 hay samples provided by clients using an aerosol monitor (DustTrak DRX 8534) in the HS (HS‐PMx) and the BZ (BZ‐PMx) of 50 horses (26 healthy, 24 asthmatic) during hay feeding. Linear regression analysis evaluated the relationship between HS‐PMx and ln BZ‐PMx for each fraction, accounting for environmental (humidity, temperature) and individual factors (horse characteristics and feeding behaviour, assessed with the Hay Contact Score).

**Results:**

The HS generated measurable dust across all PM sizes. Regression models explained 69% to 73% of ln BZ‐PMx variance, showing a weak positive association between HS‐PMx and ln BZ‐PMx across all size fractions. Hay Contact Score had the strongest positive association with ln BZ‐PMx. Cohort, ambient temperature and humidity were negatively associated with ln BZ‐PMx for certain particle sizes. The final model, incorporating HS‐PM4, Hay Contact Score, cohort, temperature, and humidity as predictors, demonstrated robust predictive accuracy for BZ‐PM4 (adjusted *R* ^2^ = 0.73).

**Main Limitations:**

Clinical impact of hay dust and poor‐quality hay was not assessed.

**Conclusions:**

The HS reliably generates hay dust for measuring standard PM fractions, particularly respirable PM4, critical to equine respiratory health. BZ dust concentrations are significantly influenced by feeding behaviour. The HS offers a standardised method for assessing hay quality, enabling informed decisions on hay selection to support respiratory health in stabled horses.

## 1 INTRODUCTION

In recent decades, a growing body of evidence has highlighted the detrimental effects of high stable dust concentrations on equine respiratory health, particularly as a triggering factor for equine asthma.^1^, ^2^, ^3^ Major sources of organic stable dust include forage^4^, ^5^, ^6^ and bedding materials,^7^, ^8^, ^9^ with concentrations influenced by specific management practices and stabling infrastructures (e.g., ventilation, cleaning, storage and feeding system).^10^, ^11^, ^12^

Stable dust consists of organic nonspecific irritants, such as bacterial (e.g., endotoxin) and fungal components (e.g., β‐glucans), as well as specific aeroallergens from moulds and mites and other antigens (e.g., latex).^13^, ^14^, ^15^ Particulate matter (PM) in stable dust can be classified by size.^3^ The inhalable fraction (<100 μm in diameter) includes all particles that can pass through the nose, encompassing the extrathoracic fraction (10–100 μm) reaching up to the larynx, the thoracic fraction (PM10, <10 μm) reaching the trachea and bronchi, the respirable fraction (PM4, <4 μm) reaching the bronchioles and the alveolar fraction (PM2.5, <2.5 μm) that penetrates to the alveoli. It is important to note that these comparisons are approximations: PM2.5 and PM10 are size fractions based on aerodynamic diameter, while inhalable, thoracic and respirable are health‐related size fractions defined by penetration efficiency determined for humans. Deposition efficiency for specific size fractions is currently not known in horses. The respirable fraction (PM4) is particularly relevant in equine asthma research due to its potential to induce lung inflammation.^16^

Two approaches are used to assess dust exposure: barn‐level and horse‐level measurements. Breathing zone (BZ) concentrations near the nostrils are expected to provide the best estimate of individual exposure and are typically higher than barn‐level concentrations.^4^, ^10^ While BZ dust concentrations are most relevant to the individual horse, they are likely influenced by the horse's behaviour during feeding and by feeding management practices (e.g., hay net vs. hay fed on the ground).^17^ Conversely, barn‐level dust measurements are affected by various factors that are challenging to standardise, such as the location of the dust measurement device, ventilation, time of day and horse density. Forage and bedding materials also significantly impact dust levels.^18^, ^19^ Given that hay is the major source of dust in barns,^3^, ^4^, ^6^ reliable assessment of its dust‐generating potential is crucial. This can help horse owners and barn managers to make informed decisions regarding their hay choices.

To standardise the evaluation of hay's dust‐generating potential, independent of horse behaviour and environmental factors, we developed the hay‐shaker (HS). This new device generates dust from hay under controlled conditions. This study aims to describe the HS and its ability to generate dust, enabling simultaneous measurement of relevant PM size fractions (PM1, PM2.5, PM4, PM10) and total dust (PMT). We also aim to compare HS measurements to BZ measurements, accounting for environmental and horse‐related factors.

## 2 MATERIALS AND METHODS

### 2.1 Study design and population

The experimental study was conducted at the Swiss Institute of Equine Medicine in Avenches, Switzerland, with data collection taking place from April to October 2023. A sample of 50 horses was enrolled, aiming for an equal distribution of asthmatic and nonasthmatic horses. Horses were classified using the Horse Owner Assessed Respiratory Signs Index (HOARSI) as either controls (HOARSI = 1) or asthmatics (HOARSI ≥2).^20^, ^21^ Only horses older than 1 year were included. Exclusion criteria were signs of systemic illness at clinical examination and any onset of respiratory distress during hay feeding. Horses were grouped into four cohorts based on their barn of origin, breed and sex. The first cohort consisted of a mix of breeds and sexes housed in the stable where the experiment was conducted. This group included horses used for leisure, sport and convalescing horses from the teaching herd. The second cohort included stallions of the Franches‐Montagnes breed housed in a nearby stable, all of which were used mainly for breeding purposes. The third cohort comprised Warmblood mares from a teaching herd kept in open‐air group housing. The fourth cohort consisted of horses brought in from outside the site, representing a variety of breeds and sexes, and included a mix of horses used for sport, leisure and breeding purposes.

For each horse, the owner provided a 5 kg sample from a recently opened hay bale from their home stable. This sample was gently manually homogenised, and two 1 kg sub‐samples were randomly selected and placed in two 110 L garbage bags. The samples were weighed using a digital luggage scale (INTERTRONIC Digital Pèse‐bagages, InterDiscount).

### 2.2 Data collection

The experiment comprised two phases: (1) measuring dust concentration generated by the HS from the first hay sample and (2) measuring dust concentration in the horse's BZ while it consumed the duplicate hay sample. Dust concentration in both phases was measured with the same device (DustTrak DRX 8534, TSI Incorporated), which provides real‐time data on airborne particulate levels across multiple size fractions (PM1, PM2.5, PM4, PM10 and total particulate matter, PMT). This device records data at one‐second intervals and is factory‐calibrated with ISO 12103‐1 A1 test dust (Arizona road dust), ensuring accurate aerosol measurements from 0.001 to 150 mg/m^3^. Airflow zero calibrations were performed before and after each experiment.

Both phases of the experiment took place in a 2.5 × 3 m enclosed area (an outdoor, roofed paddock adjacent to a stall). To minimise environmental dust contamination, the area was swept and blown clean 15 min before each experiment.

#### 2.2.1 Dust measurement in the hay shaker

**FIGURE 1:**
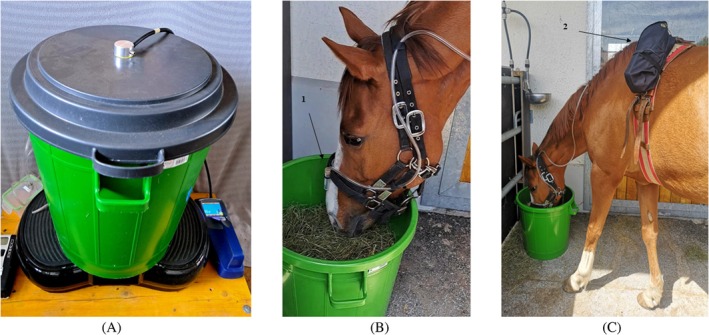
(A) Measurement of dust particles generated by the hay‐shaker (HS) equipped with the DustTrak DRX 8534 connected via a Tygon tube. (B, C) Setup for measuring dust particle concentration in the horse's breathing zone (BZ): (B) Tygon tube positioned between the nostrils with a rigid plastic tube (1) and attached to the halter; (C) DustTrak device secured in a bag (2), fastened to a lungeing girth around the thorax.

The HS (Figure 1A and Video S1) consisted of a plastic container (diameter 52 cm, height 54 cm with the cover; Landi, Dotzigen, Switzerland) mounted on a fitness vibration plate (Wellfit, 68 × 43 × 13.5 cm; Gonser) to maximise dust generation. The vibration plate was secured to a spruce wood base using threaded rods. The container lid had an aluminium and steel adapter with a 5 mm inner diameter for Tygon tube connection, while four 5 mm diameter holes in the bottom of the bucket served as air inlets. A 2 m Tygon tube connected the container to the DustTrak.

Before each use, the container was washed, sprayed with anti‐static spray (Antistatik 100 200 mL; CRC Industries Deutschland GmbH), and Tygon tubes were cleared with air to prevent cross‐contamination from previous samples and minimise environmental dust interference.

Prior to the experiment, ambient air dust concentration in the HS was measured for 2 min to establish a baseline. Afterward, 1 kg of hay was added to the container, and PM levels were recorded for 10 min during constant agitation set at the lowest vibration speed.

#### 2.2.2 Dust measurement in the horses' breathing zone

Horses were fasted for 3 h prior to the experiment to stimulate appetite. The DustTrak was secured in a bag positioned on the horse's left side and fastened to a girth around the thorax. A 2‐metre Tygon tube was connected to the sampler inlet and extended along the horse's neck, affixed to the mane from the poll down to the halter near the nostrils, where it was secured by a solid plastic tube (Figure 1B,C). Hay was presented to the horse in the same container used for the HS, without the lid. The container and Tygon tubes were cleaned as described for the HS setup. Ambient air dust concentrations were measured for 2 min prior to the experiment to establish a baseline. This was followed by a 10‐min measurement period while the horse consumed the hay. The horses were allowed to eat freely in the enclosed space and were monitored continuously to prevent interference with the measurement device. The entire measurement period was video recorded to document feeding behaviour.

#### 2.2.3 Factors with potential influence on breathing‐zone dust concentrations

Factors potentially affecting dust concentration in the horses' BZ were recorded, including horse‐related variables (cohort, feeding behaviour) and environmental conditions (ambient air temperature and humidity).

**FIGURE 2:**
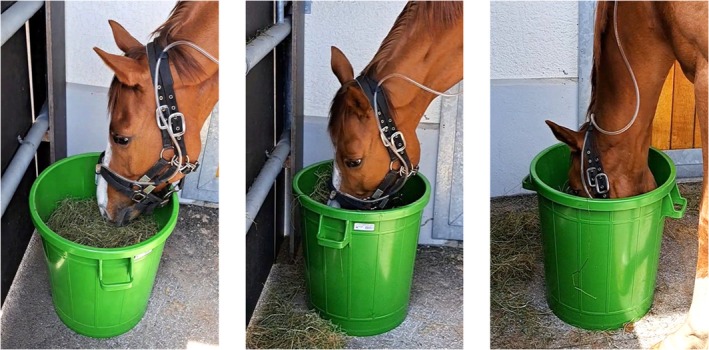
Three levels of Hay Contact Score: superficial, moderate and intense. Superficial contact involved minimal interaction (approximately 10 cm between Tygon tube and hay). Moderate contact had the Tygon tube at hay height. Intense contact buried the Tygon tube within the hay during feeding.

A subjective ‘Hay Contact Score’ was assigned to each horse based on its feeding behaviour, in a blinded manner, prior to analysing HS and BZ dust concentrations. Scoring categories (superficial, moderate or intense) reflected the predominant depth of head contact with the hay during the 10‐min feeding period (Figure 2). Superficial contact indicated minimal interaction, with only the horse's lips touching the hay, maintaining approximately 10 cm between the Tygon tube and the hay. Moderate contact occurred when the Tygon tube was positioned at hay height. Intense contact was noted when the Tygon tube remained buried within the hay throughout feeding. Additionally, the precise duration of head contact with the hay was calculated from the video recordings.

Ambient air temperature and humidity were recorded during HS and BZ measurements using a USB thermometer and hygrometer (Infactory, Pearl.ch). Measurements were taken at the start and end of each experiment (after 10 min), and average values for temperature and humidity were calculated.

### 2.3 Data processing

The TSI TrakPro software (supplied with the DustTrak DRX 8534) provided the average, minimum and maximum concentrations of airborne dust (mg/m^3^) for each particle size (PM1, PM2.5, PM4, PM10 and PMT) in spreadsheet format. For each horse, characteristics such as breed, age, sex, HOARSI, Hay Contact Score, and ambient air temperature and humidity were recorded.

## 3 DATA ANALYSES

Prior to the experiment, a power analysis was performed using G*Power Version 3.1.9.6, which determined that a sample size of 50 horses, each with its hay sample (50 hay samples), would achieve approximately 90% power, assuming an effect size between 0.33 (for 3 predictors) and 0.37 (for 5 predictors) at a significance level of ≤0.05.

Raw data were entered and cleaned in Microsoft Excel, and statistical analyses were conducted in R version 4.3.3 (R Foundation for Statistical Computing). Descriptive analyses were performed for each variable, including HS and BZ dust concentration (for each particle size), cohort (categorical), sex (categorical), breed (categorical), age (numerical), weight (numerical), Hay Contact Score (categorical), time spent with head in hay (numerical), ambient air temperature (numerical) and humidity (numerical).

Correlation coefficients were calculated between these variables: Pearson's for continuous variables and Kendall's for categorical variables.

Linear regression models were used to evaluate which of the potential predictors could contribute to the explanation of the horse's BZ (BZ‐PMx, where *x* = 1, 2.5, 4, 10 or Total). Since the outcome variables BZ‐PMx did not meet normal distribution criteria per the Shapiro–Wilk test, they were ln‐transformed to approximate normality. Preliminary analysis showed that the ‘cohort’ variable effectively captured individual characteristics (breed and sex), with a strong association between sex and cohort. To reduce the number of predictors and minimise confounding, only the cohort variable was retained in the regression model. Hay Contact Score was significantly correlated with time spent with the head in the hay (*p* < 0.05); thus, time spent in hay was excluded from the final model to avoid redundancy. The final model predictors included PM concentrations in HS (HS‐PMx where *x* = 1, 2.5, 4, 10 or Total), cohort, Hay Contact Score, ambient air temperature and humidity. A significance level of 0.05 was used for all statistical tests. Separate linear regression models were developed for each particle size fraction. Homoscedasticity was assessed using the Breusch–Pagan test, and residuals were plotted against leverage to identify potential outliers.

The final model was used to predict BZ‐PM4 based on the HS‐PM4 for each level of the Hay Contact Score, while all other predictors were fixed. PM4 was prioritised due to its relevance in equine asthma research, as particles of this size can penetrate to the bronchioles and alveoli. Cohort was fixed at level 4, which best represented the general Swiss equine population, encompassing a range of barns of origin, breeds and ages. Temperature was fixed at 22°C and Humidity at 62% as representative ambient conditions, reflecting the overall median values observed in our study.

## 4 RESULTS

### 4.1 Descriptive statistics

Raw data are provided in Tables [Link], [Link].

#### 4.1.1 Horse‐related factors

No horses were excluded due to systemic illness or respiratory distress when exposed to the hay samples. The study included 50 horses (Table S1), comprising 26 controls and 24 horses with reported signs of equine asthma. The population consisted of 25 mares, 17 geldings and 8 stallions, with ages ranging from 3 to 31 years and weights between 277 kg and 680 kg. The breeds represented were 26 Warmbloods, 13 Franches‐Montagnes, 4 ponies, 2 Quarter Horses, 2 Thoroughbreds, 2 Arabians and 1 Standardbred. The study population included 16 leisure horses, 12 sport or racehorses, 9 breeding horses and 13 horses from the teaching herd. Horses originated from 17 different stables, grouped into four cohorts: 7 horses from cohort 1, 7 from cohort 2, 11 from cohort 3 and 25 from 14 external stables (grouped as cohort 4).

The Hay Contact Score showed a balanced distribution, with 17 horses in each of the superficial and moderate contact groups, and 16 in the intense contact group. Time spent with the head in the hay varied from 81 s (14% of experiment duration) to 435 s (73% of experiment duration) with an average of 219 s (37% of experiment duration).

#### 4.1.2 Environmental factors

The ambient air temperature and humidity recorded for the 50 horses ranged from 7.1°C to 39.0°C and from 28% to 89%, respectively.

#### 4.1.3 Measurement of dust generated with the hay shaker

Each hay sample was measured at 1‐s intervals during the 10‐min experiment, yielding 600 data points per particle size. The baseline dust concentration measured during the 2‐min period prior to adding hay to the HS remained consistently lower than during the experiment (maximal values of all measurements PM1 ≤ 0.07 mg/m^3^, PM2.5 ≤ 0.08 mg/m^3^, PM4 ≤ 0.11 mg/m^3^, PM10 ≤ 0.15 mg/m^3^, PMT ≤0.15 mg/m^3^) indicating minimal carry‐over contamination between measurements and was therefore excluded from further analysis.

**FIGURE 3:**
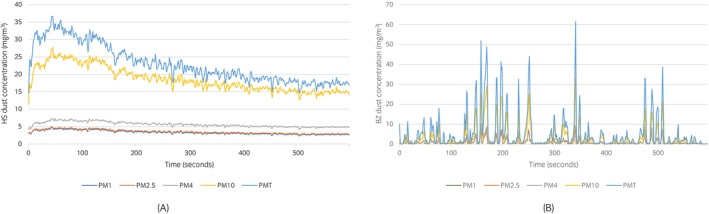
(A) Airborne dust concentration curves (mg/m^3^) for Particulate Matter (PM) PM1, PM2.5, PM4, PM10 and total dust PMT as a function of time over the 10‐min experiment duration (0–600 s), measured in the hay‐shaker (HS) for hay sample 1; (B) Airborne dust concentration curves (mg/m^3^) for PM1, PM2.5, PM4, PM10 and total dust PMT as a function of time over the 10‐min experiment duration (0–600 s), measured in the horse's breathing zone (BZ) for hay sample 1.

Table 1 presents the mean concentrations obtained for each particle size. Figure 3A illustrates the concentration curves for each particle size, using hay sample 1 as an example; similar curves were observed across all samples. All PM concentration curves displayed a rapid, steady increase within the first 3 min, followed by a gradual decline. Temporal variations were most pronounced in the PM10 and PMT fractions.

**TABLE 1:** Summary statistics of the hay‐shaker particulate matter concentration (mg/m^3^) for the 50 hay samples tested.

	PM1	PM2.5	PM4	PM10	PMT
Minimum	0.64	0.76	1.06	2.14	2.48
Maximum	8.74	9.69	13.71	38.55	46.82
Median	3.62	3.98	5.89	14.91	17.73
Mean	4.17	4.72	6.71	15.86	19.07
95% CI	[3.52–4.83]	[4.00–5.45]	[5.69–7.73]	[13.18–18.54]	[15.79–22.35]

Abbreviations: CI, confidence interval; PMx, particulate matter concentration with *x* representing the fraction size in μ, or total dust.

#### 4.1.4 Measurement of dust concentration in the breathing zone

As with the HS measurements, 600 data points per particle size were obtained for each horse over the 10‐min feeding experiment. Baseline dust concentrations measured before contact with hay were consistently lower than during the experiment (maximal values of all measurements PM1 ≤ 0.19 mg/m^3^, PM2.5 ≤ 0.20 mg/m^3^, PM4 ≤ 0.21 mg/m^3^, PM10 ≤ 0.29 mg/m^3^, PMT ≤0.50 mg/m^3^) indicating minimal environmental dust contamination and were thus excluded from the analysis.

Dust concentrations for each particle size fraction in the BZ are presented in Table 2. Unlike the stable curves observed with the HS, the BZ dust concentration curves showed significant variability across all particle sizes (illustrated in Figure 3A,B). Low background dust levels were punctuated by sharp peaks which corresponded to the moments of contact with hay. These peaks varied in frequency and intensity, influenced by the Hay Contact Score, with some overlap between peaks of different PM sizes. Figure 3B illustrates a typical pattern, though peak number and magnitude differed notably between individual horses.

**TABLE 2:** Summary statistics of the horses' breathing zone particulate matter concentration (mg/m^3^) for the 50 horses tested with their respective hay sample.

	PM1	PM2.5	PM4	PM10	PMT
Minimum	0.03	0.03	0.04	0.07	0.10
Maximum	2.35	2.44	2.77	6.05	9.40
Median	0.26	0.28	0.33	0.74	1.13
Mean	0.58	0.60	0.69	1.37	1.97
95% CI	[0.39–0.76]	[0.41–0.79]	[0.48–0.91]	[0.95–1.78]	[1.35–2.58]

Abbreviations: CI, confidence interval; PMx, particulate matter concentration with *x* representing the fraction size in μ, or total dust.

#### 4.1.5 Correlation analyses

Continuous variables (BZ‐PMx, HS‐PMx, Temperature and Humidity) were analysed using Pearson correlations. Table S2 presents the correlation coefficients and *P*‐values for these variables. Strong positive correlations were observed between the different HS‐PM fractions, with coefficients ranging from r = 0.93 to r = 1.00 (*p* < 0.001). Similarly, in the BZ, PM fractions showed correlations ranging from r = 0.9 to r = 1 (*p* < 0.001), indicating a close relationship across PM sizes. Other variable correlations were generally nonsignificant, except for a significant negative correlation between temperature and air humidity (r = −0.72, *p* < 0.001).

**FIGURE 4:**
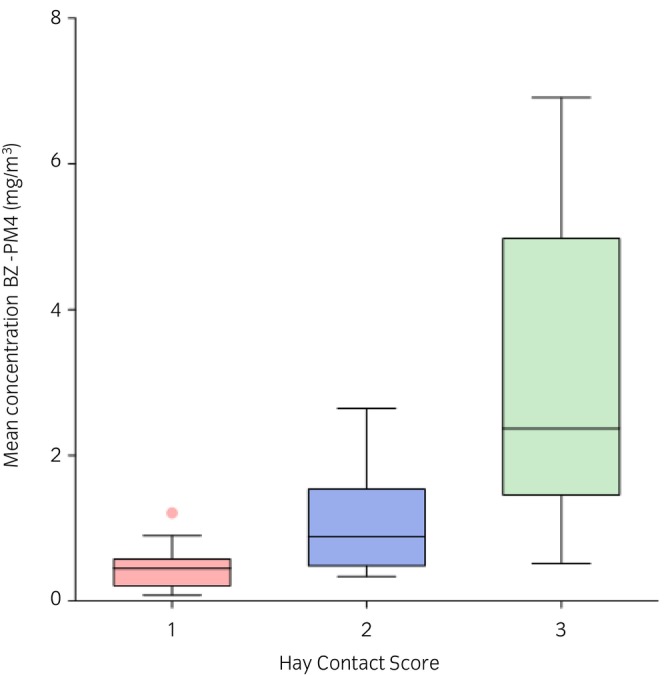
Box‐and‐whiskers plot of mean particulate matter PM4 dust concentrations (mg/m^3^) in the horses' breathing zone (BZ) by Hay Contact Score (1: superficial; 2: moderate; 3: intense).

The categorical variables (cohort and Hay Contact Score) were analysed using Kendall's correlations (Table S3). Cohort was not significantly correlated with BZ‐PMx. In contrast, the Hay Contact Score demonstrated a strong positive correlation with BZ‐PMx, ranging from r = 0.60 to r = 0.67 (*p* < 0.001), indicating that more intense hay contact corresponds to higher mean dust concentration in the BZ. Figure 4 shows BZ‐PM4 (mg/m^3^) grouped by Hay Contact Score, with similar patterns observed across all PM fractions.

### 4.2 Linear regression modelling

The final linear regression model for all PM levels was:

ln BZ‐PMx ~ HS‐PMx + Hay Contact Score + Cohort + Temperature + Humidity.

The Breusch–Pagan test indicated no heteroscedasticity, and residuals versus leverage plots revealed no significant outliers, confirming the model's robustness. Based on the adjusted R2 the final model could explain 69% to 73% of the variance in ln(BZ‐PMx) depending on the PM level.

Table 3 presents the final models for the five ln(BZ‐BM) levels. For each particle size fraction, BZ‐PMx concentration was positively associated with HS‐PMx concentration, with stronger relationships observed for smaller PM fractions (PM1 and PM2.5) compared with larger fractions. Hay Contact Score emerged as the most influential predictor in the model across all PM sizes Cohort was negatively associated with BZ dust concentrations for PM1, PM2.5 and PMT. Ambient air temperature showed a negative association with BZ‐PM levels, significant for all size fractions except for PMT, while humidity showed a significantly negative association with PM4, PM10 and PMT, but not with PM1 and PM2.5.

**TABLE 3:** Regression coefficients (*β*) for the linear regression models represent the relationship between the natural logarithm of particulate matter concentration in the breathing zone (ln BZ‐PMx) and the particulate matter generated by the hay‐shaker (HS‐PMx) for the 50 horses tested with their respective hay.

	ln BZ‐PM1	ln BZ‐PM2.5	ln BZ‐PM4	ln BZ‐PM10	ln BZ‐PMT
Intercept	−1.31	−1.30	−1.16	−0.15	0.53
95% CI	−3.22 to 0.60	−3.18 to 0.57	−3.01 to 0.69	−2.16 to 1.85	−1.51 to 2.56
*p*‐value	0.2	0.2	0.2	0.9	0.6
HS‐PMx	0.11	0.10	0.08	0.03	0.02
95% CI	0.03 to 0.19	0.03 to 0.18	0.02 to 0.13	0.01 to 0.05	0.00 to 0.04
*p*‐value	0.01*	0.008**	0.005**	0.02*	0.02*
Cohort	−0.17	−0.17	−0.16	−0.17	−0.21
95% CI	−0.34 to −0.01	−0.33 to −0.01	−0.31 to 0.01	−0.34 to 0.00	−0.39 to −0.04
*p*‐value	0.04*	0.04*	0.06	0.05	0.02*
Hay Contact Score	1.17	1.16	1.15	1.10	1.06
95% CI	0.95 to 1.39	0.95 to 1.38	0.94 to 1.37	0.87 to 1.33	0.83 to 1.30
*p*‐value	9.1e−14***	6.73e−14***	5.74e−14***	1.9e−12***	7.62e−12***
Temperature	−0.05	−0.05	−0.05	−0.04	−0.04
95% CI	−0.09 to −0.01	−0.08 to −0.01	−0.08 to −0.01	−0.08 to −0.00	−0.08 to 0.00
*P*‐value	0.02*	0.02*	0.02*	0.05*	0.06
Humidity	−0.02	−0.02	−0.02	−0.02	−0.02
95% CI	−0.04 to 0.00	−0.04 to 0.00	−0.04 to −0.00	−0.04 to −0.00	−0.04 to −0.00
*P*‐value	0.06	0.06	0.05*	0.03*	0.02*
*R* ^2^	0.75	0.75	0.76	0.73	0.72
Adjusted *R* ^2^	0.72	0.72	0.73	0.69	0.69

*Note*: HS‐PMx refers to the concentration of particulate matter generated by the hay‐shaker for different particle sizes. The subscript ‘x’ represents the specific particle size fraction, corresponding to the same size fraction listed in the BZ‐PM columns. *R* ^2^ = coefficient of determination.

Abbreviations: BZ‐PM, breathing zone particulate matter concentration; CI, confidence interval; HS‐PM, hay‐shaker particulate matter concentration.

*** *p* < 0.001;

** *p* < 0.01;

* *p* < 0.05.

These findings indicate that Hay Contact Score is the strongest predictor of dust levels in the BZ, followed by HS dust concentrations, while cohort, temperature and humidity influenced BZ dust levels only for specific size fractions.

**FIGURE 5:**
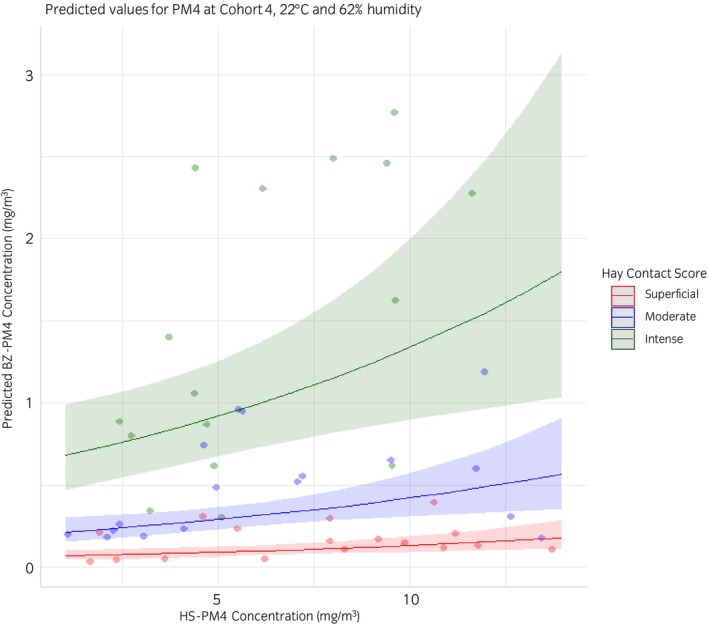
Predicted mean particulate matter PM4 in the breathing zone (BZ‐PM4) concentrations (lines) and 95% confidence intervals (coloured bands) based on measured mean particulate matter PM4 in the hay‐shaker (HS‐PM4) concentration (dots) at 22°C and 62% humidity, grouped by Hay Contact Score (red: superficial; blue: moderate; green: intense) for the 50 horses tested with their respective hay samples.

The final model was used to predict BZ‐PM4 based on HS‐PM4 in cohort 4 at 22°C and 62% humidity. Predicted values are shown in three groups according to the Hay Contact Score, the most influential predictor in the model with a semipartial correlation of 0.82 (Figure 5). The Hay Contact Score then explains 67% of BZ‐PM4, unlike HS‐PM4 (semipartial correlation 0.37), which explains only 14% of BZ‐PM4 if all other predictors remain constant. Table S4 presents the predicted mean BZ‐PM4 values based on HS‐PM4 measurements ranging from 1 to 14 mg/m^3^, which represent the minimum and maximum mean values observed across the experiments. In the final model for ln(BZ‐PM4) R‐squared was 0.76 and adjusted R‐squared 0.73.

## 5 DISCUSSION

This study introduces a novel method for standardised dust generation from hay samples and compares dust concentrations measured with the HS to those measured in the horses' BZ. The HS proved to be a practical and robust tool, allowing simultaneous measurement of 5 PM size fractions using the DustTrak. Baseline values, measured following thorough cleaning of the HS, were consistently lower than those during the experiment, confirming that sequential testing of different hay samples without cross‐contamination is possible. Various types of equipment have previously been used to assess the dustiness of different feeds and bedding materials.^8^, ^22^, ^23^ Here we present a novel method for standardised dust measurements from agitated hay samples without environmental or horse influence.

Our second aim was to compare the dust measurements from the HS (HS‐PMx) to those obtained in the horses' BZ (BZ‐PMx), accounting for environmental and horse‐related factors during hay feeding. The HS generated approximately ten times more dust than was observed in the horses' BZ. This difference was expected since the HS is a closed system with continuous agitation throughout the measurement period, thereby generating more dust than typical horse feeding behaviour, which is characterised by intermittent and variable contact with hay.

Dust concentrations in the BZ varied considerably among individual horses. To estimate daily exposure levels from our measurements, we calculated a range based on published data indicating that horses typically spend 25% to 52% of the day eating.^24^, ^25^, ^26^ This approach allowed us to estimate the minimum and maximum daily PM2.5 and PM10 exposure levels. For PM2.5, daily exposure ranged from 0.0075 to 1.27 mg/m^3^, and for PM10, from 0.02 to 3.15 mg/m^3^. These BZ concentrations markedly exceed the recommended thresholds for human exposure. The World Health Organisation advises daily exposure limits of 0.015 mg/m^3^ for PM2.5 and 0.045 mg/m^3^ for PM10 for short‐term exposure (24 h, 3–4 days per year), and limits of 0.005 and 0.015 mg/m^3^, respectively, for annual daily exposure.^27^ Since exceeding these limits has been shown to adversely affect lung health in humans, the elevated BZ dust levels present potential risks to equine respiratory health.^28^ Although no equivalent recommendations exist for short‐ or long‐term dust exposures in horses, our findings can be contextualised with several studies that have used different methods to investigate airborne dust levels in stabled horses.

Dust concentrations measured in the BZ during the 10‐min feeding period, particularly for PM4, aligned most closely with results from a study that also reported total dust values during hay feeding.^29^, ^30^ In contrast, other studies examining BZ dust concentration values in horse stables under various management systems report values that are considerably (up to 10 times) higher^10^, ^14^ or lower.^5^, ^17^, ^31^ A recent study found lower dust concentrations for PM2.5 and higher values for PM10 in the BZ.^32^ Notably, PM10 concentrations in the BZ during hay feeding were significantly higher in this study compared with those reported by Millerick‐May et al.,^33^ who used the same type of real‐time monitor and methodology but fed hay on the ground. This wide range of reported dust concentrations is likely attributable to differences in study designs, measurement methodologies and equipment, hay type and quality and other dust sources. In this study, feeding hay in a deep tub likely contributed to the higher dust concentrations observed, resembling conditions associated with ‘slow‐feeders’. This setup was chosen to reduce background variability when hay was fed on the ground. Additionally, one study mentions substantial individual variability in dust exposure among horses housed in the same stable environment.^17^ However, previous research has not examined how horse behaviour during hay feeding may impact BZ dust concentrations.

In the present study, the Hay Contact Score emerged as the strongest predictor of BZ dust concentrations, highlighting the role of individual feeding behaviour. Horses that maintained closer contact with the hay, especially those with immersing their heads deeply in the hay container, exhibited significantly higher BZ‐PMx levels, particularly for PM4—a key fraction for respiratory health. This finding suggests that BZ dust measurements reflect feeding behaviour more than the intrinsic dustiness of the hay. Ivester et al.^17^ previously demonstrated that area measurements in stables tend to underestimate and poorly correlate with BZ dust exposure, proposing an ‘equine personal cloud effect’ driven by individual behaviour. The concept of a ‘personal dust cloud’ is also used in human respiratory environmental medicine to explain variations in individual dust exposures influenced by personal activities.^34^, ^35^

The Hay Contact Score provides a practical means of applying the personal dust cloud concept in horses, offering a subjective measure of hay contact intensity. Notably, we found a strong association between the Hay Contact Score and the time horses spent with their heads in contact with hay during the experiment. This straightforward assessment thus allows horses to be grouped by feeding behaviour without the need for precise time recording, making it a useful tool for future studies. Its simplicity also makes it accessible for use by horse owners and caretakers, enabling them to identify feeding habits that may increase dust exposure.

In contrast to feeding behaviour, the association between BZ dust concentrations and horse cohort was weak. The cohort variable was selected to represent intrinsic factors such as sex, breed and barn of origin. For example, cohort 2 consisted exclusively of Franches‐Montagnes stallions housed in a single barn, which may have distinct behaviours and routines compared with other groups. However, this association was significant only for PM1, PM2.5 and PM10. These findings suggest that individual horse behaviour has a far greater influence on dust exposure than other intrinsic factors.

Our study also identified a weak negative association between the environmental factors of ambient temperature and humidity and BZ dust concentrations. Specifically, colder and drier conditions were related to higher dust concentrations. This finding aligns with a previous study that reported similar effects of ambient air temperature and humidity on dust formation in horse stables.^36^ In contrast, another study found a positive correlation between ambient temperature and PM4 in the barn and no association with humidity.^17^ However, the overall influence of these factors was limited. Humidity showed a significant association only with larger particles (BZ‐PM4, BZ‐PM10 and BZ‐PMT), suggesting that higher humidity reduces the aerosolisation of larger dust fractions. A recent review similarly reported that relative humidity was mostly negatively associated with particles larger than PM2.5, while positively correlated with finer particles (PM2.5 and smaller).^37^

Overall, the present results demonstrate that hay dust measurements in the BZ and in the HS provide complementary insights: BZ measurements are highly sensitive to individual horses' feeding behaviour, while the HS provides a standardised assessment of dust generation from hay samples.

Combining our findings, the predictive model offers valuable estimates of the dust exposure risk in horses based on feeding behaviour and hay dustiness. For instance, a horse with a superficial Hay Contact Score feeding on hay with low HS‐PM4 levels (1 mg/m^3^) shows a very low predicted mean BZ‐PM4 of just 0.07 mg/m^3^. In contrast, a moderate Hay Contact Score combined with moderate HS‐PM4 levels (7 mg/m^3^) predicts an approximately five‐fold higher BZ‐PM4 of 0.34 mg/m^3^. With an intense Hay Contact Score and high HS‐PM4 levels (14 mg/m^3^), the predicted BZ‐PM4 rises another five‐fold to 1.80 mg/m^3^. This model underscores the critical role of both individual feeding behaviour and hay dustiness in managing dust exposure.

## 6 LIMITATIONS

Although the experiments were rigorously conducted, employing complementary approaches to compare HS and BZ hay dust measurements, this study has certain limitations. First, clinical data on the horses' respiratory health were not included in the analyses. Historical information on respiratory signs (HOARSI) was used solely to ensure that the study sample included both clinically healthy horses and those showing asthma‐like clinical signs. This study was not designed to assess the effect of dust concentrations on lung health; however, incorporating clinical evaluations in future studies could provide valuable insights into the clinical impact of dust exposure. This could be achieved by combining standardised assessment of hay dustiness (HS) and personal dust cloud evaluations (Hay Contact Score) with established respiratory health assessments, such as bronchoalveolar lavage cytology and lung function testing.

Second, the short experimental duration may not fully capture horses' daily dust exposure. Dust measurements were taken over a 10‐min period, during which individual feeding behaviour varied considerably. A recent study found that BZ dust levels can be overestimated when measured over short intervals (20 min) compared with extended periods (8 h).^32^ However, the variation factor remained consistent when comparing dry hay to soaked hay, leading the authors to conclude that the first 20 min of feeding provide a reliable estimate of overall dust exposure. Future studies could extend the observation period to assess the impact of feeding behaviour on long‐term dust exposure in the BZ. Likewise, repeated HS measurements across multiple samples from the same hay source could provide further insights into hay quality variability.

Third, this study evaluated only moderate‐ to good‐quality hay, as deemed suitable for consumption by owners and caretakers. This may not capture the full spectrum of particulate matter concentration variation, as poor‐quality, mouldy hay might generate significantly more dust. Evaluating the HS device's ability to assess a broader range of hay qualities, as well as various forage types (e.g., haylage, hay, straw), hay varieties (e.g., alfalfa, timothy grass), hay treatments (e.g., soaking, steaming) and bedding materials (e.g., wood shavings, straw) would be valuable. The HS could prove to be a very useful tool for investigating the impact of different management practices on air quality in horse stables.

Lastly, the calibration of the dust measurement device using Arizona Dust, while a standard practice, presents some limitations. Arizona Dust may not fully replicate the chemical and physical properties of stable dust. However, its use ensures comparability with other studies, as it is widely used for dust calibration across various environments. Future work could consider more stable‐specific calibration to improve accuracy in equine settings. A recent study^32^ highlighted that this type of instrument may introduce significant biases when measuring organic dust, such as hay dust. Consequently, comparisons with PM exposure determined by gravimetric methods, considered the reference standard, should be interpreted with caution unless an appropriate correction factor is applied. However, relative comparisons, such as those between the measurements in the HS and in the BZ, remain valid and appropriate.

## 7 CONCLUSIONS

These findings highlight the complexity of assessing particulate matter exposure in equine environments. Direct comparisons indicate that BZ dust concentrations are primarily influenced by feeding behaviour, whereas the HS provides a standardised assessment of hay dust levels in a controlled setting. This novel device offers an effective means of evaluating hay dustiness without animal exposure to inhaled dust. Combined with the practical Hay Contact Score, HS measurements allow for reliable predictions of dust exposure levels. Together, the BZ and HS dust measurements provide complementary perspectives to investigate and optimise husbandry in horse stables, ultimately supporting equine respiratory health.

## FUNDING INFORMATION

Internal Research Fund of the Swiss Institute of Equine Medicine, Bern, Switzerland (ISMEquine Research No. 33‐890). This study was also supported by an animal welfare foundation based in Geneva, Switzerland, which wishes to remain anonymous.

## CONFLICT OF INTEREST STATEMENT

The authors declare no conflicts of interest.

## AUTHOR CONTRIBUTIONS

**Virginie Marie Angèle Bouverat:** Writing – original draft; conceptualization; investigation; methodology. **Jan Naef:** Conceptualization; methodology; writing – review and editing. **Gaudenz Dolf:** Formal analysis; software; writing – review and editing. **Inès Lamon:** Resources; methodology. **Sophie Elena Sage:** Writing – review and editing; supervision; validation. **Vinzenz Gerber:** Writing – review and editing; supervision; conceptualization; funding acquisition; methodology; validation.

## DATA INTEGRITY STATEMENT

Virginie Marie Angèle Bouverat had full access to all the data in the study and takes responsibility for the integrity of the data and the accuracy of data analysis.

## ETHICAL ANIMAL RESEARCH

The study was approved by the Animal Experimentation Committee of the Canton of Vaud, Switzerland, under permit number VD3861.

## INFORMED CONSENT

Informed written consent was obtained from the horse owners.

## Supporting information


**Table S1.** Population characteristics grouped by cohort. For continuous variables, the mean (minimum, maximum) is reported.

**Table S2.** Pearson correlation coefficients between dust measurements and other numerical variables, along with their *p* values.

**Table S3.** Kendall's tau b correlation coefficients between dust measurements in the Breathing Zone, Cohort and Hay Contact Score, along with their *p*‐values.

**Table S4.** Predicted mean BZ‐PM4 concentrations based on the measured mean HS‐PM4 concentration (mg/m^3^) at a temperature of 22°C and a humidity of 62%, by Hay Contact Score (superficial, moderate and intense).

**Video S1.** Hay‐shaker in operation during experiments.

## Data Availability

The data that support the findings of this study are openly available in Zenodo at https://doi.org/10.5281/zenodo.14789742, reference number [14789742].

## References

[1] Couëtil LL , Cardwell JM , Gerber V , Lavoie J‐P , Léguillette R , Richard EA . Inflammatory airway disease of horses—revised consensus statement. J Vet Intern Med. 2016;30(2):503–515. 10.1111/jvim.13824 26806374 PMC4913592

[2] Ivester KM , Couëtil LL , Zimmerman NJ . Investigating the link between particulate exposure and airway inflammation in the horse. J Vet Intern Med. 2014;28(6):1653–1665. 10.1111/jvim.12458 25273818 PMC4895611

[3] Art T , McGorum BC , Lekeux P . Environmental control of respiratory disease. US, Ithaca NY: International Veterinary Information Service; 2002 [cited 2024]. Available from: https://www.ivis.org/library/equine-respiratory-diseases/environmental-control-of-respiratory-disease

[4] Auger E‐J , Moore‐Colyer MJS . The effect of management regime on airborne respirable dust concentrations in two different types of horse stable design. J Equine Vet Sci. 2017;51:105–109. 10.1016/j.jevs.2016.12.007

[5] Olave CJ , Ivester KM , Couetil LL , Kritchevsky JE , Tinkler SH , Mukhopadhyay A . Dust exposure and pulmonary inflammation in standardbred racehorses fed dry hay or haylage: a pilot study. TVJ. 2021;271:105654. 10.1016/j.tvjl.2021.105654 33840486

[6] Olave CJ , Ivester KM , Couëtil LL , Franco‐Marmolejo J , Mukhopadhyay A , Robinson JP , et al. Effects of forages, dust exposure and proresolving lipids on airway inflammation in horses. Am J Vet Res. 2021;83(2):153–161. 10.2460/ajvr.21.08.0126 34843444

[7] Burrell MH , Wood JL , Whitwell KE , Chanter N , Mackintosh ME , Mumford JA . Respiratory disease in thoroughbred horses in training: the relationships between disease and viruses, bacteria and environment. Vet Rec. 1996;139(13):308–313. 10.1136/vr.139.13.308 8893488

[8] Fleming K , Hessel EF , van den Weghe H . Generation of airborne particles from different bedding materials used for horse keeping. J Equine Vet Sci. 2008;28(7):408–418. 10.1016/j.jevs.2008.05.004

[9] Mönki J , Saastamoinen M , Karikoski N , Rajamäki M , Raekallio M , Junnila J , et al. Effects of bedding material on equine lower airway inflammation: a crossover study comparing peat and wood shavings. Front Vet Sci. 2021;8:656814. 10.3389/fvets.2021.656814 33898547 PMC8062776

[10] Woods PS , Robinson NE , Swanson MC , Reed CE , Broadstone RV , Derksen FJ . Airborne dust and aeroallergen concentration in a horse stable under two different management systems. Equine Vet J. 1993;25(3):208–213. 10.1111/j.2042-3306.1993.tb02945.x 8508749

[11] Wålinder R , Riihimäki M , Bohlin S , Hogstedt C , Nordquist T , Raine A , et al. Installation of mechanical ventilation in a horse stable: effects on air quality and human and equine airways. Environ Health Prev Med. 2011;16(4):264–272. 10.1007/s12199-010-0195-5 21431789 PMC3117214

[12] Webster AJ , Clarke AF , Madelin TM , Wathes CM . Air hygiene in stables. 1: effects of stable design, ventilation and management on the concentration of respirable dust. Equine Vet J. 1987;19(5):448–453. 10.1111/j.2042-3306.1987.tb02641.x 3678188

[13] Douwes J , Thorne P , Pearce N , Heederik D . Bioaerosol health effects and exposure assessment: progress and prospects. Ann Occup Hyg. 2003;47(3):187–200. 10.1093/annhyg/meg032 12639832

[14] Samadi S , Wouters IM , Houben R , Jamshidifard A‐R , van Eerdenburg F , Heederik DJJ . Exposure to inhalable dust, endotoxins, beta(1–3)‐glucans, and airborne microorganisms in horse stables. Ann Occup Hyg. 2009;53(6):595–603. 10.1093/annhyg/mep040 19561032

[15] Wyler M , Sage SE , Marti E , White S , Gerber V . Protein microarray allergen profiling in bronchoalveolar lavage fluid and serum of horses with asthma. J Vet Intern Med. 2023;37(1):328–337. 10.1111/jvim.16600 36479920 PMC9889601

[16] Pearson CC , Sharples TJ . Airborne dust concentrations in livestock buildings and the effect of feed. J Agri Engin Res. 1995;60(3):145–154. 10.1006/jaer.1995.1008

[17] Ivester KM , Smith K , Moore GE , Zimmerman NJ , Couëtilt LL . Variability in particulate concentrations in a horse training barn over time. Equine Vet J. 2012;43:51–56. 10.1111/j.2042-3306.2012.00647.x 23447878

[18] Crichlow EC , Yoshida K , Wallace K . Dust levels in a riding stable. Equine Vet J. 1980;12(4):185–188. 10.1111/j.2042-3306.1980.tb03422.x 7439142

[19] Clements JM , Pirie RS . Respirable dust concentrations in equine stables. Part 1: validation of equipment and effect of various management systems. Res Vet Sci. 2007;83(2):256–262. 10.1016/j.rvsc.2006.12.002 17477944

[20] Ramseyer A , Gaillard C , Burger D , Straub R , Jost U , Boog C , et al. Effects of genetic and environmental factors on chronic lower airway disease in horses. J Vet Intern Med. 2007;21:149–156. 10.1111/j.1939-1676.2007.tb02941.x 17338163

[21] Laumen E , Doherr MG , Gerber V . Relationship of horse owner assessed respiratory signs index to characteristics of recurrent airway obstruction in two warmblood families. Equine Vet J. 2010;42(2):142–148. 10.2746/042516409X479586 20156250

[22] Hessel EF , Garlipp F , van den Weghe HF . Generation of airborne particles from horse feeds depending on type and processing. J Equine Vet Sci. 2009;29(9):665–674. 10.1016/j.jevs.2009.07.013

[23] Herholz C , Kocher J , Küng P . Pferdegesundheit: Staub‐ und Ammoniakemissionenvon acht verschiedenen Einstreumaterialien. Agrarforschung Schweiz. 2020;11:230–237. 10.34776/afs11-230

[24] Harris PA , Ellis AD , Fradinho MJ , Jansson A , Julliand V , Luthersson N , et al. Review: feeding conserved forage to horses: recent advances and recommendations. Animal. 2017;11(6):958–967. 10.1017/S1751731116002469 27881201

[25] Hart R , Bailey A , Farmer J , Duberstein K . Chewing analysis of horses consuming bermudagrass hay in different styles of slow feeders as compared to loose hay. J Equine Vet Sci. 2024;140:105133. 10.1016/j.jevs.2024.105133 38908808

[26] Ellis A . Biological basis of behaviour in relation to nutrition and feed intake in horses. EAAP Sci Ser. 2010;128:53–74.

[27] La Salud OM de, World Health Organization . WHO global air quality guidelines: particulate matter (PM2.5 and PM10), ozone, nitrogen dioxide, sulfur dioxide and carbon monoxide. Geneva: World Health Organization; 2021. 34662007

[28] Elfman L , Riihimäki M , Pringle J , Wålinder R . Influence of horse stable environment on human airways. J Occup Med Toxicol. 2009;4:10. 10.1186/1745-6673-4-10 19467158 PMC2693518

[29] Bartz J , Hartung J . Dust measurements on a horse using an “equine personal sampler”. Livestock Environment IV: 4th International Symposium. St. Joseph, MI: American Society of Agricultural Engineers; 1993 ;742–746. 10.5555/19932460163

[30] McGorum BC , Ellison J , Cullen RT . Total and respirable airborne dust endotoxin concentrations in three equine management systems. Equine Vet J. 1998;30(5):430–434. 10.1111/j.2042-3306.1998.tb04514.x 9758102

[31] Clements JM , Pirie RS . Respirable dust concentrations in equine stables. Part 2: the benefits of soaking hay and optimising the environment in a neighbouring stable. Res Vet Sci. 2007;83(2):263–268. 10.1016/j.rvsc.2006.12.003 17467753

[32] Ivester KM , Ni J‐Q , Couetil LL , Peters TM , Tatum M , Willems L , et al. A wearable real‐time particulate monitor demonstrates that soaking hay reduces dust exposure. Equine Vet J. 2025;57(4):1065–1073. 10.1111/evj.14425 39463012 PMC12135757

[33] Millerick‐May ML , Karmaus W , Derksen FJ , Berthold B , Robinson NE . Airborne particulates (PM10) and tracheal mucus: a case–control study at an American thoroughbred racetrack. Equine Vet J. 2015;47(4):410–414. 10.1111/evj.12303 24905487

[34] Ferro AR , Kopperud RJ , Hildemann LM . Elevated personal exposure to particulate matter from human activities in a residence. J Expo Anal Environ Epidemiol. 2004;14(Suppl 1):S34–S40. 10.1038/sj.jea.7500356 15118743

[35] Delfino RJ , Quintana PJE , Floro J , Gastañaga VM , Samimi BS , Kleinman MT , et al. Association of FEV1 in asthmatic children with personal and microenvironmental exposure to airborne particulate matter. Environ Health Perspect. 2004;112(8):932–941. 10.1289/ehp.6815 15175185 PMC1242025

[36] Herholz C , Kocher J , Küng P , Burren A . Digital monitoring of dust release in a horse stable, depending on ventilation opening area and bedding type. PHK. 2020;36(4):316–324. 10.21836/PEM20200405

[37] Tanatachalert T , Jumlongkul A . Correlation between relative humidity and particulate matter during the ongoing of pandemic: a systematic review. Aerosol Sci Eng. 2023;7(3):295–302. 10.1007/s41810-023-00186-5

