# Supplementary material for: Minimising feeding behaviour interference: A hay‐shaker device to assess dust exposure in horses
Source: Equine Vet J. 2025 Mar 3;57(6):1666–76. doi: 10.1111/evj.14492 (PMC12508277; doi:10.1111/evj.14492)
Supplement: Supplementary file 1 — Table S1. Population characteristics grouped by cohort. For continuous variables, the mean (minimum, maximum) is reported. [file EVJ-57-1666-s005.pdf]

Equine Veterinary Journal BEVA

Table S1: Population characteristics grouped by cohort. For continuous variables, mean (minimum, maximum) are reported.

Variable		Cohort 1	Cohort 2	Cohort 3	Cohort 4
**N**		7	7	11	25
**Sex**	Mare	2 (29%)	0 (0%)	11 (100%)	12 (48%)
	Gelding	4 (57%)	0 (0%)	0 (0%)	13 (52%)
	Stallion	1 (14%)	7 (100%)	0 (0%)	0 (0%)
**Age (years)**		9 (3, 13)	14 (4, 31)	12 (6, 20)	13 (5, 23)
**Weight (kg)**		561 (445, 680)	509 (478, 570)	575 (512, 630)	520 (277, 671)
**Breed**	Warmblood	4 (57%)	0 (0%)	10 (91%)	12 (48%)
	Franches-Montagnes	0 (0%)	7 (100%)	1 (9%)	5 (20%)
	Pony	0 (0%)	0 (0%)	0 (0%)	4 (16%)
	Quarter Horse	1 (14%)	0 (0%)	0 (0%)	1 (4%)
	Thoroughbred	2 (29%)	0 (0%)	0 (0%)	0 (0%)
	Arabian	0 (0%)	0 (0%)	0 (0%)	2 (8%)
	Standardbred	0 (0%)	0 (0%)	0 (0%)	1 (4%)
**Purpose**	Leisure	2 (29%)	0 (0%)	0 (0%)	14 (56%)
	Sport / Race	3 (43%)	0 (0%)	0 (0%)	9 (36%)
	Breeding	0 (0%)	7 (100%)	0 (0%)	2 (8%)
	Teaching Herd	2 (29%)	0 (0 %)	11 (100%)	0 (0%)
**HOARSI**	1	5 (71%)	0 (0%)	11 (100%)	10 (40%)
	2 – 4	2 (29%)	7 (100%)	0 (0%)	15 (60%)
**Hay Contact Score**	Superficial	2 (29%)	3 (43%)	6 (55%)	6 (24%)
	Moderate	3 (43%)	0 (0%)	3 (27%)	11 (44%)
	Intense	2 (29%)	4 (57%)	2 (18%)	8 (32%)

Abbreviations: N, number of horses; HOARSI, Horse Owner Assessed Respiratory Signs Index.
