# Supplementary material for: Minimising feeding behaviour interference: A hay‐shaker device to assess dust exposure in horses
Source: Equine Vet J. 2025 Mar 3;57(6):1666–76. doi: 10.1111/evj.14492 (PMC12508277; doi:10.1111/evj.14492)
Supplement: Supplementary file 2 — Table S2. Pearson correlation coefficients between dust measurements and other numerical variables, along with their p values. [file EVJ-57-1666-s003.pdf]

Table S2: Pearson correlation coefficients between dust measurements and other numerical variables, along with their *P*-values.

	HS-PM1	HS-PM2.5	HS-PM4	HS-PM10	HS-PMT	BZ-PM1	BZ-PM2.5	BZ-PM4	BZ-PM10	BZ-PMT	Temp	Hum
**HS-PM1**	**1.00**	**1.00**	**1.00**	**0.96**	**0.95**	**0.14**	**0.14**	**0.14**	**0.13**	**0.13**	**0.25**	**-0.18**
P-value	0.00	<0.001	<0.001	<0.001	<0.001	0.34	0.33	0.34	0.37	0.37	0.08	0.22
**HS-PM2.5**	**1.00**	**1.00**	**1.00**	**0.94**	**0.93**	**0.14**	**0.14**	**0.14**	**0.13**	**0.12**	**0.26**	**-0.18**
P-value	<0.001	0.00	<0.001	<0.001	<0.001	0.32	0.33	0.32	0.37	0.40	0.07	0.22
**HS-PM4**	**1.00**	**1.00**	**1.00**	**0.96**	**0.95**	**0.13**	**0.14**	**0.14**	**0.13**	**0.13**	**0.26**	**-0.20**
P-value	<0.001	<0.001	0.00	<0.001	<0.001	0.34	0.34	0.34	0.37	0.38	0.07	0.17
**HS-PM10**	**0.96**	**0.94**	**0.96**	**1.00**	**1.00**	**0.12**	**0.12**	**0.12**	**0.14**	**0.16**	**0.20**	**-0.19**
P-value	<0.001	<0.001	<0.001	0.00	<0.001	0.42	0.41	0.42	0.35	0.27	0.17	0.18
**HS-PMT**	**0.95**	**0.93**	**0.95**	**1.00**	**1.00**	**0.12**	**0.12**	**0.11**	**0.13**	**0.16**	**0.19**	**-0.18**
P-value	<0.001	<0.001	<0.001	<0.001	0.00	0.42	0.43	0.43	0.35	0.26	0.19	0.21
**BZ-PM1**	**0.14**	**0.14**	**0.13**	**0.12**	**0.12**	**1.00**	**1.00**	**0.99**	**0.93**	**0.90**	**0.05**	**-0.11**
P-value	0.34	0.33	0.35	0.42	0.43	0.00	<0.001	<0.001	<0.001	<0.001	0.74	0.46
**BZ-PM2.5**	**0.14**	**0.14**	**0.14**	**0.12**	**0.12**	**1.00**	**1.00**	**1.00**	**0.94**	**0.91**	**0.05**	**-0.11**
P-value	0.33	0.32	0.34	0.41	0.42	<0.001	0.00	<0.001	<0.001	<0.001	0.71	0.45

**BZ-PM4**	**0.14**	**0.14**	**0.14**	**0.12**	**0.11**	**0.99**	**1.00**	**1.00**	**0.96**	**0.93**	**0.06**	**-0.13**
P-value	0.34	0.32	0.34	0.42	0.43	<0.001	<0.001	0.00	<0.001	<0.001	0.67	0.39
**BZ-PM10**	**0.13**	**0.13**	**0.13**	**0.14**	**0.13**	**0.93**	**0.94**	**0.96**	**1.00**	**0.98**	**0.07**	**-0.19**
P-value	0.37	0.37	0.37	0.35	0.35	<0.001	<0.001	<0.001	0.00	<0.001	0.64	0.18
**BZ-PMT**	**0.13**	**0.12**	**0.13**	**0.16**	**0.16**	**0.90**	**0.91**	**0.93**	**0.98**	**1.00**	**0.07**	**-0.24**
P-value	0.37	0.40	0.38	0.27	0.26	<0.001	<0.001	<0.001	<0.001	0.00	0.65	0.10
**Temp**	**0.25**	**0.26**	**0.26**	**0.20**	**0.19**	**0.05**	**0.05**	**0.06**	**0.07**	**0.07**	**1.00**	**-0.72**
P-value	0.08	0.07	0.07	0.17	0.19	0.74	0.71	0.67	0.64	0.65	0.00	<0.001
**Hum**	**-0.18**	**-0.18**	**-0.20**	**-0.19**	**-0.18**	**-0.11**	**-0.11**	**-0.13**	**-0.19**	**-0.24**	**-0.72**	**1.00**
P-value	0.22	0.22	0.17	0.18	0.21	0.46	0.45	0.39	0.18	0.10	<0.001	0.00

Abbreviations: BZ-PM, Breathing Zone Particulate Matter concentration; HS-PM, Hay-Shaker Particulate Matter concentration; Temp: ambient air temperature; Hum: ambient air humidity.
