# Supplementary material for: Minimising feeding behaviour interference: A hay‐shaker device to assess dust exposure in horses
Source: Equine Vet J. 2025 Mar 3;57(6):1666–76. doi: 10.1111/evj.14492 (PMC12508277; doi:10.1111/evj.14492)
Supplement: Supplementary file 3 — Table S3. Kendall's tau b correlation coefficients between dust measurements in the Breathing Zone, Cohort and Hay Contact Score, along with their p‐values. [file EVJ-57-1666-s001.pdf]

Equine Veterinary Journal BEVA

Table S3: Kendall's tau b correlation coefficients between dust measurements in the Breathing Zone, Cohort and Hay Contact Score, along with their *P*-values.

Variable	BZ-PM1	BZ-PM2.5	BZ-PM4	BZ-PM10	BZ-PMT	Cohort	Hay Contact Score
**Cohort**	**-0.08**	**-0.07**	**-0.07**	**-0.13**	**-0.16**	**1.00**	**0.06**
P-value	0.46	0.51	0.51	0.25	0.14	<0.001	0.64
**Hay Contact Score**	**0.67**	**0.67**	**0.67**	**0.63**	**0.60**	**0.06**	**1.00**
P-value	<0.001	<0.001	<0.001	<0.001	<0.001	0.64	<0.001

Abbreviations : BZ-PM, Breathing Zone Particulate Matter concentration.
