# Supplementary material for: Minimising feeding behaviour interference: A hay‐shaker device to assess dust exposure in horses
Source: Equine Vet J. 2025 Mar 3;57(6):1666–76. doi: 10.1111/evj.14492 (PMC12508277; doi:10.1111/evj.14492)
Supplement: Supplementary file 4 — Table S4. Predicted mean BZ‐PM4 concentrations based on the measured mean HS‐PM4 concentration (mg/m3) at a temperature of 22°C and a humidity of 62%, by Hay Contact Score (superficial, moderate and intense). [file EVJ-57-1666-s004.pdf]

Equine Veterinary Journal BEVA

Table S4: Predicted mean BZ-PM4 concentrations based on the measured mean HS-PM4 concentration (mg/m^3) at a temperature of 22°C and a humidity of 62%, by Hay Contact Score (superficial, moderate and intense).

	Superficial		Moderate		Intense	
**Measured HS-PM4**	**Predicted BZ-PM4**	**95% CI**	**Predicted BZ-PM4**	**95% CI**	**Predicted BZ-PM4**	**95% CI**
**1**	0.07	0.04, 0.10	0.22	0.15, 0.30	0.68	0.47, 0.99
**2**	0.07	0.05, 0.11	0.23	0.17, 0.31	0.74	0.52, 1.04
**3**	0.08	0.06, 0.11	0.25	0.19, 0.33	0.79	0.57, 1.10
**4**	0.09	0.06, 0.12	0.27	0.21, 0.34	0.85	0.62, 1.17
**5**	0.09	0.07, 0.13	0.29	0.23, 0.37	0.92	0.67, 1.26
**6**	0.1	0.07, 0.13	0.31	0.25, 0.39	0.99	0.72, 1.36
**7**	0.11	0.08, 0.14	0.34	0.27, 0.42	1.07	0.77, 1.48
**8**	0.11	0.08, 0.16	0.36	0.28, 0.47	1.15	0.82, 1.63
**9**	0.12	0.09, 0.17	0.39	0.30, 0.51	1.24	0.86, 1.80
**10**	0.13	0.09, 0.19	0.42	0.31, 0.57	1.34	0.90, 1.99
**11**	0.14	0.10, 0.21	0.46	0.32, 0.64	1.44	0.93, 2.22
**12**	0.16	0.10, 0.23	0.49	0.33, 0.72	1.55	0.97, 2.49
**13**	0.17	0.11, 0.26	0.53	0.35, 0.81	1.67	1.00, 2.79
**14**	0.18	0.11, 0.29	0.57	0.36, 0.91	1.8	1.04, 3.13

Abbreviations : BZ-PM, Breathing Zone Particulate Matter concentration; CI, Confidence Interval; HS-PM, Hay-Shaker Particulate Matter concentration.
